# Birth preparedness and complication readiness practice and influencing factors among women in Sodo town, Wolaita zone, Southern Ethiopia, 2018; community based cross-sectional study

**DOI:** 10.1186/s12978-019-0703-z

**Published:** 2019-03-29

**Authors:** Gedion Asnake Azeze, Taklu Marama Mokonnon, Melkamu Worku Kercho

**Affiliations:** Department of Midwifery, College of Health Science, Wolaita Sodo University, P.O.Box 138, Sodo, Ethiopia

**Keywords:** Birth preparedness, Counseling, Ethiopia, Practice

## Abstract

**Background:**

Birth preparedness and complication readiness (BPCR) is a strategy that helps women to consider all available maternal health care services during pregnancy and prepare for potential complications. Though global annual number of maternal deaths decreased to an estimated 303,000 in 2015, avoidable morbidity and mortality remains a formidable challenge in many developing countries which account for approximately 99% (302,000) of the global maternal deaths in 2015. This study aims to assess the practice and factors associated with birth preparedness and complication readiness among women in Sodo town, Wolaita zone, Southern Ethiopia; 2018.

**Methods:**

Community-based cross-sectional study was carried out from June 1–30, 2018. A total of 495 (pregnant and recently delivered women), were randomly selected and interviewed using pretested structured questionnaire. The data were entered using EPI Data version 3.1 and analyzed using SPSS version 20. Descriptive statistics were reported and bivariate and multivariable logistic regression was carried out to see the effect of each independent variable on the dependent variable.

**Result:**

Of 506 sampled participants, 495 (49.5% pregnant and 50.5% recently delivered) participated, which made a response rate of 97.8%. 48.5% of women were prepared for birth and ready for its complication in the study area. From multivariable analysis, women in the age group of 15–24 (AOR = 2.39, 95% C. I = 1.19, 4.46) and 25–34 years (AOR = 1.89, 95% C. I = 1.10, 3.25); women who attended college and above level of education (AOR = 2.07, C. I = 1.11, 3.88); women counseled to prepare potential blood donors (AOR = 1.90, 95% C. I = 1.15, 3.12) and to identify skilled birth attendants prior to birth (AOR = 1.59, 95% C. I = 1.05, 2.39) and women whose partners and/or families were counseled (AOR = 2.16, 95%C.I = 1.25, 3.74) were factors positively associated with birth preparedness and complication readiness practice.

**Conclusion:**

Although not satisfactory in view of expectations, a relatively higher practice of birth preparedness and complication readiness had been observed in the study area compared with the previous reports. Healthcare workers at the grassroots should be encouraged to involve women’s partners and/or family members while explaining birth preparedness and complication readiness with a special emphasis on older (> 35 years) and uneducated women in order to improve the practice in the study area.

## Plain English summary

Birth preparedness and complication readiness are interventions designed to address the delays in seeking, reaching and obtaining care by encouraging pregnant women, their families, and communities to effectively plan for births and prepare for emergencies if they occur. Studies conducted in low- and middle-income countries revealed that, promoting birth preparedness and complication readiness improves preventive behavior and knowledge of mothers about danger signs thereby leading to improvement in care-seeking during obstetric emergency. However, avoidable maternal morbidity and mortality remains a formidable challenge in many developing countries due to the poor practice of birth preparedness and complication readiness. Therefore, the aim of this study is to explore the practice and influencing factors of preparedness for birth and readiness for its complication. The study was conducted in Sodo town, Wolaita zone, Ethiopia from June 1–30, 2018. Simple random sampling technique was used to select Kebeles (Lowest administrative units) and study participants. Data were collected by face to face interview technique using a structured questionnaire. Strength of association was determined using multivariable logistic regression model. Accordingly, age, educational status, counseled to prepare potential blood donor in case of emergency, counseled to identify skilled birth attendant ahead of childbirth, counseling given to partner and/or family members about BPCR during ANC visits. Hence, healthcare workers at the grassroots should also be encouraged to involve women’s partners and/or family members while explaining birth preparedness and complication readiness with a special emphasis on older (> 35 years) and uneducated women in order to improve the practice in the study area.

## Background

Since child birth is unpredictable risk producing event, providing timely and adequate medical care for women who experience obstetric complication is an option for mitigating the risk. Identifying and reducing delays in seeking, reaching and obtaining care are the key strategies for timely use of skilled maternal and neonatal care, especially during the time of pregnancy, labor and child birth. This can be achieved by one of the essential component of globally accepted safe motherhood programs; Birth Preparedness and Complications Readiness (BPCR), a strategy that helps women to consider all available maternal health care services during pregnancy and prepare for potential complications [1, 2].

Birth preparedness and complication readiness is among the key interventions with the objective to reduce maternal mortality through promoting health care seeking behavior and utilization of appropriate heath care facilities and skilled personnel for delivery [3]. The World Health Organization (WHO) recommends that pregnant women should receive focused antenatal care in which birth preparedness and complication readiness is a key component [4]. It is advised that birth plans are discussed at the booking visit, then reviewed in subsequent visits and finalized by 32 weeks [5]. BPCR promotes active preparation and decision making for birth among women and their families and encourages to identify a skilled birth attendant (SBA); identifying the location of the closest appropriate care facility; arranging transport to a health facility for birth and obstetric emergencies; identify two compatible blood donors, save funds for birth-related and emergency expenses; identify of a person to take care of the family in case of the expectant mother’s absence; prepare important and any needed supplies for mother at birth as well as supplies for postnatal care, being aware of the expected date of delivery and on the key obstetrics danger signs [6].

Though global annual number of maternal deaths decreased to an estimated 303,000 in 2015, avoidable morbidity and mortality remains a formidable challenge in many developing countries which account for approximately 99% (302,000) of the global maternal deaths in 2015. Likewise, the lifetime risk of maternal mortality is estimated at 1 in 36 in sub-Saharan Africa, contrasting sharply with approximately 1 in 4900 in developed countries [7]. This enormous discrepancy highlights one of the most striking aspect of maternal mortality: its hugely disproportionate burden on poor countries [8]. Beside the risk of dying, WHO estimated 300 million women are suffering from short term or long term illnesses that occur during pregnancy and labor [9].

Sustainable Development Goals (SDGs) establish a transformative new agenda for maternal health towards ending preventable maternal mortality; target 3.1 of SDG 3 is to reduce the global Maternal Mortality Rate (MMR) to less than 70 per 100,000 live births by 2030. Achieving this global goal will require countries to reduce their MMR by at least 7.5% each year between 2016 and 2030, more than three times the 2.3% annual rate of reduction observed globally between 1990 and 2015. The SDG also establishes a supplementary national target that no country should have an MMR greater than 140 per 100,000 live births [7, 10].

The government of Ethiopia launched free delivery services at any public health facilities beginning in 2005 with the aim of saving maternal and newborn lives by encouraging more women to deliver their babies in health facilities [11]. However, as evidenced by Demographic Health Survey dataset, MMR in Ethiopia has not reduced; for instance, the 2005 Ethiopian Demographic Health Survey (EDHS) report showed MMR of 673 per 100,000 livebirths but it gets increased by 3, while it was expected to be decreased greatly, and becomes 676 per 100,000 livebirths in the following 2011 report [12, 13]. Though the MMR decreased to 412/100,000 live births in 2016 EDHS report [14], far more work is needed to achieve substantially higher annual rates of reduction to attain MMRs below 140 in 2030. As birth preparedness and complication readiness is one of the basic strategy set to reduce MMR, the purpose of this study was therefore, to assess the practice and factors affecting birth preparedness and complication readiness among pregnant women to their first 12 months postpartum preceding the survey in Sodo town, Wolaita zone, Ethiopia.

## Methods and material

### Study setting and design

Community based cross sectional study was employed in Sodo town, Wolaita zone, South Nation Nationality of People Regional State, Ethiopia from June 1–30, 2018. Sodo town is located 390 km south of Addis Ababa, the capital city of Ethiopia. The town is divided in to 4 administrative sub-city. The total population of the town in 2018, received from the town administrative office, was 182,607 (93,130 males and 89,477 females) from these, 4963 were women in the reproductive age group (15-49 years). There are also 28,499 under-five age children and 4576 infants less than one year of age. Functioning health facilities in the town includes two Hospitals (one governmental and one private), 3 health centers, 17 medium and lower level clinics and 17 health posts.

### Source and study population

The source of population for this study were all women found in the reproductive age group (15–49 years) living in Sodo town, Wolaita zone, Southern Ethiopia. The potential study population comprised from a list of second trimester (> 28 weeks of gestational age) pregnant women to their first 12 months postpartum preceding the survey obtained from community health extension workers (CHEWs) in each kebelles of the respective sub-city. For the pregnant participants, the inclusion criteria were; pregnant women beyond second trimester (> 28 weeks gestational age), have had at least 2 antenatal visits, and permanent resident of the study area. The second categories of participants were women who recently delivered (delivered within 12 months prior to the study). Women who were mentally disabled and severely ill were excluded.

### Sample size determination and sampling procedure

Sample size was calculated using the single population proportion formula by taking the following considerations: The proportion (p) of BPCR taken from previously conducted study in the region [15], the margin of error or precision (w), and confidence level were assumed to be 18.3, 5, and 95%, respectively. Considering a design effect of 2 and a non-response rate of 10%, the final sample size was 506. Then, the total sample size was divided equally in to two; the first half (*n* = 253) for currently pregnant women and the remaining half for women who have had delivered in the last 12 months preceding the survey.

The study sampling was carried out in a small community with 12 rural and 3 urban woreda and with a total of 345 kebeles, smallest local administrative units in the city. Multistage sampling was used to select the study participants. First, all the Kebeles were stratified in to urban and rural. Then, the total sample size was allocated proportionally to the size of the selected kebeles. Finally, systematic sampling was employed to select the study participants in each kebele until the desired numbers of sample was obtained. The first household was selected by simple random sampling; lottery method. Then, the next house hold was selected through systematic sampling technique that is every K^th^ interval household which was calculated for each kebele. In a case when the study participants were not able to be interviewed for some reason (e.g. absenteeism), three visit rule was made to interview the respondent and after all, they were considered as non-respondents.

### Measurements

Data was collected by face to face interview technique using a structured questionnaire. The questionnaire was taken from the safe mother hood questionnaire developed by maternal and neonatal health program of JHPIEGO, the affiliate of Johns Hopkins University [16] and this was further complimented with variables from related studies and adapted according to local context and the objectives of the study. It was prepared in English then translated into Amharic and Wolaitegna versions and back-translated to check for internal consistency. The questionnaire covered socio demographics, education/counseling on BPCR components during ANC visits, Knowledge of obstetric and neonatal key danger signs and participants’ practice of birth preparedness and complication readiness. Six Community Health Extension Workers (CHEWs) form the neighborhood town (Areka town) interviewed the eligible pregnant women and they were supervised by two Bachelor of Science (BSc.) Nursing holders. Data collectors and supervisors were oriented and trained for two day*s* on how to interview and record the data before the start of the survey.

### Data processing and analysis

The filled questionnaires were checked for completeness and entered into Epi Data version 3.1 and transported to SPSS window version 20 for analysis. Descriptive statistics were done. Both bivariate and multivariable logistic regression models were used to identify associated factors. All variables significantly associated with practice of birth preparedness and complication readiness at less than or equals to 0.25 *p*-values in the bivariable logistic regression model were fitted into the multivariable logistic regression model to control the effect of confounding variables. Odds ratios and their 95% CIs were computed and variables with p - value less than 0.05 were considered statistically significant.

### Data quality assurance

Data quality was controlled by giving trainings and appropriate supervisions for data collectors. Data collectors and supervisors were local language speakers. A pre-test was conducted on 5% of the questionnaire on one of unselected kebeles and based on a pretest result, additional adjustment was made. Appropriate modifications were made after analyzing the pretest result before the actual data collection.

#### Operational and term definitions

Birth preparedness and complication ready: A woman was considered as prepared for birth and its complication if she identified place of delivery; skilled birth attendants; saved funds for birth-related and emergency expenses, arranged transport to a health facility for birth and obstetric emergencies, and arranged blood donor in cases of emergency. These five variables were transformed on SPSS into a single variable that is ‘Birth Preparedness and Complication Readiness Practice’. Women who followed at least three of the five BPCR components were considered as “prepared for birth and its complication” and coded as 1. The remaining women were considered as “not prepared for birth and its complication” and coded as 0. This scoring has been previously used in studies that assessed women’s BPCR practice [1, 3, 8, 17, 18].

### Ethical considerations

Ethical clearance was obtained from institutional review board of Wolaita Sodo University, College of Health Sciences. Official letter of permission was written to the respective study sub-cities and administrative office at the selected Keble’s were communicated through formal letters. Participants were informed about the purpose, benefit, risk, confidentiality of information and the voluntary nature of participation in the study. Participants were informed that they had the right to withdraw from the study at any time and also, informed verbal consent was obtained from participants before conducting the interview.

## Result

### Sociodemographic characteristics of participants

A total of 506 women were identified to participate in the study. Out of these 495 were interviewed making a response rate of 97.8%. The mean and ± Standard Deviation (SD) of respondent age was 28.74 ± 6.14 years. Wolaita were the predominant ethnic group, 387 (78.2%), followed by Amhara 38(7.7%), Gurage 28(5.7), Oromo 25(5.1%) and the rest (Tigray, Sidama and Hadiya) 17 (3.4%) were categorized as ‘others’. The majority of participants’ family income 381(67.8%) were less than 500 Ethiopia Birr with the mean and ± Standard Deviation of 2385.86 ± 1749.59. 295 (59.6%), 178 (36.0), 22(4.4%) of the study participants had ≤4, 5–6 and 7 or more family size respectively with the median of 4 and interquartile range (IQR) of 4.0 (Table 1).

**Table 1:** Sociodemographic characteristics of women of reproductive age group, Wolaita Sodo town, Wolaita zone, Ethiopia; 2018 (*n* = 495)

Variables	Birth Preparedness and Complication Readiness	
	No Frequency (%)	Yes Frequency (%)
Age of the respondent		
15–24	50 (42.7)	67 (57.3)
25–34	132 (50.0)	132 (50.0)
35–44	73 (64.0)	41 (36.0)
Religion		
Protestant	159 (52.8)	142 (47.2)
Orthodox	63 (48.8)	66 (51.2)
Muslim	15 (42.9)	20 (57.1)
Catholic	15 (65.2)	8 (34.8)
Others^a^	3 (42.9)	4 (57.1)
Marital status		
Married	193 (50.4)	190 (49.6)
Single	28 (56.0)	22 (44.0)
Divorced	13 (50.0)	13 (50.0)
Widowed	15 (62.5)	9 (37.5)
Cohabiting	6 (50.0)	6 (50.0)
Educational status of the women		
No formal education	69 (64.5)	38 (35.5)
Primary education (1–8)	112 (49.3)	115 (50.7)
Secondary education (9–12)	36 (57.1)	27 (42.9)
College and above	38 (38.8)	60 (61.2)
Husband/Partner’s educational status		
No formal education	28 (58.3)	20 (41.7)
Primary education (1–8)	104 (50.5)	102 (49.5)
Secondary education (9–12)	59 (54.6)	49 (45.4)
College and above	64 (48.1)	69 (51.9)
Women’s occupational status		
Housewife	146 (57.7)	107 (42.3)
Merchant	39 (45.9)	46 (54.1)
Daily laborer	35 (54.7)	29 (45.3)
Government employee	23 (44.2)	29 (55.8)
Private employee	9 (33.3)	18 (66.7)
Student	3 (21.4)	11 (78.6)
Husband/Partner’s occupation		
Farmer	24 (47.1)	27 (52.9)
Merchant	78 (48.8)	82 (51.2)
Daily laborer	70 (60.3)	46 (39.7)
Government employee	51 (47.2)	57 (52.8)
Private employee	19 (52.8)	17 (47.2)
Student	13 (54.2)	11 (45.8)
Monthly family income		
< 500	16 (51.6)	15 (48.4)
500–1000	118 (52.4)	107 (47.6)
> 1000	121 (50.6)	118 (49.4)

^a^ 7th Day Adventist, Traditional

### Oxbstetrics characteristics of the participants

Regarding obstetric characteristics of the participants; ninety eight (19.8%) women got pregnant only once and the highest gravida (total number of pregnancy) was six with median and inter quartile range (IQR) of 2.0 (the IQR showed that 50% of the women were pregnant for 2–4 times). Among the study participants 480 (97.0%) of the women had no history of still births while 24 (4.8%) reported that they had history of abortion (Table 2).

**Table 2:** Obstetric characteristics of women of reproductive age group, Wolaita Sodo town, Wolaita zone, Ethiopia; 2018 (*n* = 495)

VARIABLES	Frequency (n)	Percentage (%)
Maternal status		
Pregnant	245	49.5
Recently delivered	250	50.5
First pregnancy		
Yes	95	19.8
No	397	80.2
Total N^o^ of pregnancy		
Primigravida (1)	98	19.8
Multigravida (2–4)	361	72.9
Grand multigravida (≥5)	36	7.3
Parity		
Nulliparous (0)	21	4.2
Primiparous (1)	139	28.1
Multiparous (2–4)	311	62.8
Grand multiparous (≥5)	24	4.8
Planned/unplanned pregnancy		
Unplanned	155	31.3
planned	340	68.7
ANC visits for current pregnancy		
< 4	353	71.3
≥ 4 visits	142	28.7
Counseled on where to go if health problems happen		
No	327	66.1
Yes	168	33.9
Counseled to arrange blood donors		
No	403	81.4
Yes	92	18.6
Counseled on saving funds		
No	335	67.7
Yes	160	32.3
Counseled on identifying SBA		
No	338	68.3
Yes	157	31.7
Partners and/or Family counseled on BPCR		
No	411	83.0
Yes	84	17.0
Discussion with partner about BPCR		
No	199	40.2
Yes	296	59.8
Counseled on Arrangement of transportation		
No	213	43.0
Yes	282	57.0
Counseled on place of delivery		
No	95	19.2
Yes	400	80.8
History of family planning use		
No	129	26.1
Yes	366	73.9
History of abortion		
Yes	24	4.8
No	471	95.2
History of stillbirth		
Yes	15	3.0
No	480	97.0
Previous pregnancy/childbirth complications		
Yes	50	10.1
No	445	89.9
Partners and/or families counseled about BPCR		
No	359	72.5
Yes	136	27.5
Discussion about BPCR with partner’s		
No	148	29.9
Yes	347	70.1
Partner’s plan for place of delivery		
Not planned	96	19.4
Home	31	6.3
Health institution	368	74.3

*SBA* Skill Birth Attendance

### Knowledge of key obstetric and neonatal danger signs

**Fig. 1:**
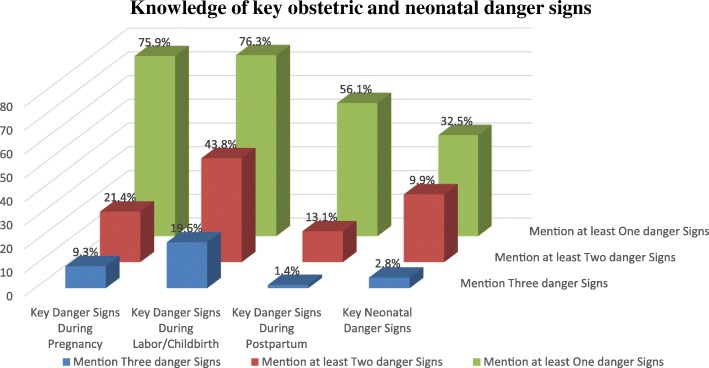
Knowledge of key obstetric and neonatal danger signs among women of reproductive age group, Wolaita Sodo town, Wolaita zone, Ethiopia; 2018 (n = 495)

Sever vaginal bleeding was the commonest key danger sign mentioned by 73.5, 67.7 and 54.9% during pregnancy, childbirth and postpartum period respectively. Other possible or potential key dangers that were mentioned include; swollen hand (15.8%), blurred vision (8.1%), and water breaks without labor (12.1%) were mentioned during pregnancy; labor lasting more than 12 h (34.1%), convulsion during labor (4.8%) and retained placenta for more than 30 min (33.9%) mentioned during childbirth; high fever (11.7%) and foul smelling vaginal discharge (4.0%) during the postpartum period. 84.8, 49.3, and 23.0% of the participants were able to mention at least one, two and three key danger signs respectively in all the three phases. Concerning to knowledge of key neonatal danger signs, difficult/fast breathing, lethargy, convulsion/spasm and very small baby were mentioned by 28.7, 8.9, 5.1, and 3.4% of the study participants respectively (Fig. 1).

Our study finding showed that around 9.3, 19.6, and 13.1% of the participants were able to mention at least a total of three (two for the later) key danger signs and considered as knowledgeable on key obstetric danger signs during pregnancy, childbirth and postpartum respectively while, only 2.8% of participants mentioned at least three out of four neonatal key danger sign and considered as knowledgeable.

### Birth preparedness and complication readiness practice of participants

The birth preparedness score was computed from key elements of birth preparedness and complication readiness components. By considering 3 or more steps of the five components, 240 (48.5%) were found to fulfill the criteria and considered as prepared for birth and its complications (Table 3).

**Table 3:** Birth preparedness and complication readiness practice of women of reproductive age group, Wolaita Sodo town, Wolaita zone, Ethiopia; 2018 (*n* = 495)

Component of Birth Preparedness and Complication Readiness Items	Frequency (n)	Percent (%)
Plan for place of birth		
No	149	30.1
Yes	346	69.9
Identified SBA to assist at birth		
No	284	57.4
Yes	211	42.6
Arranging transportation		
No	305	61.6
Yes	190	38.4
Preparing blood donors		
No	487	98.4
Yes	8	1.6
Saved Funds		
No	65	13.1
Yes	430	86.9
Number of steps taken	Frequency (n)	Percent (%)
Unable to mention any component	10	2.0
Mention at least one component	485	98.0
Mention at least two component	408	82.4
Mention at least three component	240	48.5
Mention at least four component	52	10.5
Mention all five components	None	

*SBA* Skill Birth Attendant

### Factors affecting practice of birth preparedness and complication readiness

In bivariate analysis, the variables found to be associated with BPCR at *p*-value < 0.25 were: age, educational status, occupational status, previous history of family planning utilization, whether the current pregnancy was planned or unplanned, gravidity, parity, total number of ANC visits, counseled to prepare blood donors, counseled to identify skilled birth attendant, counseling given to partners and or family members about BPCR, having discussion with partners about BPCR at home, knowledge regarding key danger signs during pregnancy and during childbirth. After adjusting for the potential cofounders; multivariable logistic regression analysis with backward conditional method indicated that age, educational status, counseled to prepare potential blood donor in case of emergency, counseled to identify skilled birth attendant ahead of childbirth, counseling given to partners and/or family about BPCR during ANC visits were significantly associated (*p*-value < 0.05) with the practice of BPCR.

The odds of BPCR practice were higher among those study participants in the age group of 15–24 and 25–34; where the odds of practicing BPCR were more than two-fold (AOR = 2.39, 95% C. I = 1.19, 4.46) and almost 90% more likely (AOR = 1.89, 95% C. I = 1.10, 3.25) among women in the age group of 15–24 and 25–34 years respectively than those women above 35 years. Women’s educational status was another factor significantly associated with the practice of BPCR in such, the practice were two times higher (AOR = 2.07, C. I = 1.11, 3.88) than their comparative women with no formal education.

Furthermore, the finding of this study shows that women who had received counseling during ANC visits to prepare potential blood donors in cases of obstetric complication and to identify skilled birth attendants prior to birth were 90% (AOR = 1.90, 95% C. I = 1.15, 3.12) and almost 60% (AOR = 1.59, 95% C. I = 1.05, 2.39) more likely to practice BPCR than their counterparts. Those women whose partners and/or families were counseled on BPCR were two times more likely (AOR = 2.16, 95%C.I = 1.25, 3.74) to practice than those who reported not (Table 4).

**Table 4:** Factors affecting practice of Birth Preparedness and Complication Readiness adjusted for confounding variables in Wolaita Sodo town, Wolaita zone, Ethiopia: 2018 (*n* = 495)

Variables	Birth Preparedness and Complication Readiness		Crude OR (95% C.I)	Adjusted OR (95% C.I)
	No (%)	Yes (%)		
Age				
15–24	50 (42.7)	67 (57.3)	2.39 (1.40, 4.05)	2.31 (1.19, 4.46)*
25–34	132 (50.0)	132 (50.0)	1.78 (1.13, 2.79)	1.89 (1.10, 3.25)*
35–44	73 (64.0)	41 (36.0)	1	1
Women’s educational level				
No formal education	69 (64.5)	38 (35.5)	1	1
Primary education	112 (49.3)	115 (50.7)	1.86 (1.16, 2.99)	1.56 (0.94, 2.59)
Secondary education	36 (57.1)	27 (42.9)	1.36 (0.72, 2.57)	1.07 (0.53, 2.13)
College and above	38 (38.8)	60 (61.2)	2.86 (1.62, 5.05)	2.07 (1.11, 3.88)*
Women’s Occupational status				
Housewife	146 (57.7)	107 (42.3)	0.20 (0.05, 0.73)	
Merchant	39 (45.9)	46 (54.1)	0.32 (0.08, 1.23)	
Daily laborer	35 (54.7)	29 (45.3)	0.23 (0.05, 0.88)	
Government employee	23 (44.2)	29 (55.8)	0.34 (0.08, 1.37)	
Private employee	9 (33.3)	18 (66.7)	0.55 (0.12, 2.46)	
students	3 (21.4)	11 (78.6	1	
History of family planning use				
No	78 (60.5)	51 (39.5)	1	
Yes	177 (48.4)	189 (51.6)	1.63 (1.08, 2.45)	
Gravida				
1	42 (42.9)	56 (57.1)	1	
2–4	197 (54.6)	164 (45.4)	0.62 (0.39, 0.98)	
≥ 5	16 (44.4)	20 (55.6)	0.93 (0.43, 2.02)	
Parity				
Nulliparous (0)	7 (33.3)	14 (66.7)	1	
Primiparous (1)	58 (41.7)	81 (58.3)	0.69 (0.26, 1.83)	
Multiparous (2–4)	180 (57.9)	131 (42.1)	0.36 (0.14, 0.92)	
Grand multiparous ≥5	10 (41.7)	14 (58.3)	0.70 (0.20, 2.36)	
Counseled to prepare blood donors				
No	216 (53.6)	187 (46.4)	1	1
Yes	39 (42.4)	53 (57.6)	1.57 (0.99, 2.48)	1.90 (1.15, 3.12)*
Counseled to Identifying skilled birth attendant				
No	183 (54.1)	155 (45.9)	1	1
Yes	72 (45.9)	85 (54.1)	1.39 (0.95, 2.03)	1.59 (1.05, 2.39)*
Family/Partners were counseled on BPCR				
No	228 (55.5)	183 (45.5)	1	1
Yes	27 (32.1)	57 (67.9)	2.63 (1.59, 4.32)	2.16 (1.25, 3.74)*
Discussion with partner about BPCR				
No	122 (61.3)	77 (38.7)	1	
Yes	133 (44.9)	163 (55.1)	1.94 (1.34, 2.79)	
Total number of ANC visits				
< 4 Visits	193 (54.7)	160 (45.3)	1	
≥ 4 Visits	62 (43.7)	80 (56.3)	1.56 (1.05, 2.30)	
Pregnancy planned/unplanned				
Unplanned	68 (43.9)	87 (56.1)	1	
Planned	187 (55.0)	153 (45.0)	0.63 (0.43, 0.93)	
Knowledge on key danger sign during pregnancy				
Not knowledgeable	238 (53.0)	211 (47.0)	1	
Knowledgeable	17 (37.0)	29 (63.0)	1.92 (1.02, 3.60)	
Knowledge on key danger sign during childbirth				
Not knowledgeable	211 (53.0)	187 (47.0)	1	
Knowledgeable	44 (45.4)	53 (54.6)	1.35 (0.87, 2.12)	

*Significant association at *p*-value < 0.05

## Discussion

### Practice of birth preparedness and complication readiness

This study was conducted among women who were pregnant or gave birth recently by taking a priori sample size calculation to determine the number of study participants and as such, the study was adequately powered as planned. A high proportion of participants (86.9%) reported that, they saved money as one of preparation for childbirth. Likewise, saved money was the most common element of BPCR in studies conducted in Ethiopia, central Tanzania, Eti-Osa local government area of Lagos State, Rwanda, and Northern Ghana [2, 3, 17, 19, 20]. The observed similarities between studies might be due to the fact that women know that having money can help them in buying important supplies or pay for transportation in case of obstetrics emergency. Making arrangement for blood donors in case of emergency, another key elements of BPCR, was mentioned by only small proportion (1.6%) of the study participants. Our study finding was in line with studies conducted in Ethiopia, Northern Ghana, and Nepal [2, 20, 21]. One possible reason for the observed similarity in finding might be due to the fact that blood transfusion is considered to be a critical condition and few women think they will reach that condition.

By considering three or more BPCR components, the overall practice of women who were well prepared for birth and ready for complication was 240 (48.5% with 95% CI of 44.0–53.0). The prevalence was lower than studies conducted in Addis Ababa, Ethiopia (56.3, 72.6%), Southwest Nigeria, (82.1%), Tanzania (58.2%), Southern province of Sri Lanka (83.5%), West Bengal (57%) and Karnataka, India (79.3%) [3, 4, 9, 18, 22–24]. The observed variation might be due to methodological difference across areas and between studies. For instance, studies conducted at Addis Ababa, Ethiopia and Sri Lanka [44, 47, 21] used exit interview to assess BPCR among pregnant women attending ANC at health facilities where facility-based study, rather than community-based, have the possibility of overestimating the result. Moreover, studies conducted at Bengal and Karnataka, India [4, 18] a relatively small sample was studied which might be responsible for the observed difference. Nevertheless, the finding of this study is congruent with study finding level of; 43.4% of Northeast Ethiopia [25], 48.4% of Edo State and Nigeria [26]. The observed similarity between these findings might be due to the fact that studies were conducted in the same country and continent and it’s believed that these countries will not have significant difference in their socio-demographic characteristics.

Our study finding was higher as compared with study conducted in Ethiopia; 23.3% of Jimma Zone [27], 29.9% of Goba woreda, Bale zone [28]. This difference could be the result of efforts and multi-sectoral collaborations that have been made by the Government of Ethiopia since 2013 on maternal, newborn and child health through Health Extension Workers (HEWs) and one-to-five networks, particularly focusing on encouraging women to deliver in health facilities which can also be witnessed by the reports of EDHS; the reduction in Maternal Mortality Rate (MMR) from 676/100,000 livebirth in 2011 to 412/100,000 livebirths in 2016 [13, 29]. However, the studies mentioned above [27, 28] used data which was collected before or nearly at the commencement of the implementation. As well, the results of this study was also higher when compared with findings conducted in; Lagos, Nigeria (33.4%) [17], and in Rwanda (22.3%) [19]. Methodological difference might be a reason for this observed variation in findings. For instance, in the aforementioned studies, participants were pregnant women and they may not able to report whether they identified/prepared for things that they have not yet needed and therefore, might be unable to share their experience regarding BPCR. We have tried to decrease this limitation by taking study participants both pregnant (to control for recall problems of women who have recently delivered) and recently delivered women (to control for likely inability of current pregnant women to adequately share their experience).

### Factors influencing practice of birth preparedness and complication readiness

The odds of practicing BPCR was higher among those women who were relatively younger; women aged 15–24 were two times (AOR = 2.39, 95% C. I = 1.19, 4.46) and those 25–34 years were 90% (AOR = 1.89, 95%C.I = 1.10, 3.25) more likely to be prepared for birth and its complication compared to those aged 35 years and above. The finding is in line with findings from Ethiopia and Southwest Nigeria [22, 30]. The possible reason is that older women who might belong to a more traditional cohort, tend to have experience in delivery and feel that they can deliver safely without the help of any health professional. However, our finding differed with findings from Karnataka, India where, women aged > 26 years were almost three times prepared for birth and ready for complications compared to younger ones [4]. The observed difference in finding might be due to older women of Karnataka, India might experience complications on their previous obstetric history (though not assessed in the study) therefore, trying to get prepared for the current pregnancy. Further, this may also be due to difference in health-seeking behavior among the study participants.

Education increases the likelihood that women will develop and implement a birth plan [31]. As well, the present study revealed two fold (AOR = 2.07, 95%C.I: 1.11, 3.88) increases in BPCR among women who attended college and above level of education than illiterate mothers. Similar findings has been observed in the study conducted in Ethiopia; preparation for birth and its complication was associated with respondent who attended secondary and above educational level than uneducated women [32]. It could be due to the fact that, education gives an opportunity to develop greater confidence, to make better choices and to make decisions regarding their own health. Moreover, women who have attained high levels of education are able to adhere to counseling provided at ANC and better understand the health messages acquired from various sources.

Counseling women about components of birth preparedness and complication readiness during ANC visit was positively associated with the practice; those women who received counseling about identifying skilled birth attendant and arranging blood donors ahead of childbirth were 60% (AOR = 1.59, 95%C.I = 1.05, 2.39) and 90% (AOR = 1.90, 95% C. I = 1.15, 3.12) more likely to be prepared for birth and it’s complications respectively compared to women who did not. There was similar study finding conducted at Adigrat town, Ethiopia in which preparation for birth and its complication was higher among those who were advised about birth preparedness during their antenatal care follow up [33]. This might be due to the fact that through focused antenatal care, women are equipped with health education and receive counseling on how to practice BPCR which may improve early decision making and care-seeking in case of delivery or obstetric emergency. Therefore, it is expected that women who attend ANC will receive counseling on BPCR and thus, ideally, there should be 100% positive agreement that counseling occurred. However, both the level and quality of counseling specifically on danger signs and pregnancy complications during ANC visits are insufficient as has been reported elsewhere [3, 34, 35].

Women who had reported their partner/family received counseling on BPCR practiced more than two times (AOR = 2.16, 95%C.I = 1.25, 3.74) as compared to women who did not. There was a related finding conducted at Karnataka, India where, woman’s discussion about BP/CR with her family members had a significant association with optimal BPCR practice [4]. Here in Ethiopia, it is not surprising to see husbands deciding the timing and conditions of sexual relations, family size and whether or not their spouses will utilize available health care services. Therefore, partner/family counseling, though a male partner is rarely seen at ANC, will enable men to support their spouses to utilize emergency obstetric services early and the couple would adequately prepare for birth and make themselves ready for complications. Further, it is believed that husbands who had counseled during ANC visit may provide financial and psychological support to their spouse.

Though the authors consider to include knowledge of neonatal key danger signs and assessing the issues addressed in ANC education/counseling while studding BPCR (not assessed in many of previously conducted studies) were considered as a strength, this study is not without limitation. First, self-reporting may have introduced reporter bias as it may be difficult validating claims provided by participants and second, its limitation is associated with not ascribing the direction of causations to the relationships found in the study because of the nature of cross sectional study design.

## Conclusion

Although not satisfactory in view of expectations, a relatively higher practice of BPCR had been observed in the study area compared with the previous reports. This study revealed that a large majority of participants saved money as one of preparation for childbirth while only few prepared blood donors in case of obstetric complication and emergency. Age, educational status, counseled to prepare potential blood donor in case of emergency, counseled to identify skilled birth attendant ahead of childbirth, counseling given to partner/family members about BPCR during ANC visits were found to be significantly associated with the practice of BPCR, calling for more interventions to be taken at health institutions and health care providers.

This finding stresses the importance of improved training for health providers on how to better communicate BPCR related messages with antenatal care attendants and the need to address additional barriers to the uptake of BPCR. Further, healthcare workers at the grassroots should also be encouraged to involve women’s partners and/or family members while explaining birth preparedness and complication readiness with a special emphasis on older (> 35 years) and uneducated women in order to improve the practice in the study area. Lastly, the authors recommend further studies focusing quality improvement research on why women prepared less, addressing many confounding variables that influence birth outcomes, the social determinants of health, access to a skilled provider, distance from facility, etc.

## Abbreviations

ANC: Antenatal Care
BPCR: Birth Preparedness and Complication Readiness
CHEW: Community health extension worker
EDHS: Ethiopian Demographic and Health Survey
MMR: Maternal Mortality Rate
SBA: Skilled birth attendant
SDG: Sustainable Development Goals
WHO: World Health Organization
